# Comparative analysis of dexmedetomidine, midazolam, and propofol impact on epilepsy-related mortality in the ICU: insights from the MIMIC-IV database

**DOI:** 10.1186/s12883-024-03693-1

**Published:** 2024-06-07

**Authors:** Xun Li, Wei Yue

**Affiliations:** 1 Clinical College of Neurology, Neurosurgery, and Neurorehabilitation, Tianjin Medical University, Tianjin, China; 2 Department of Neurology, Tianjin Huanhu Hospital, No.6 Ji Zhao Road, Jinnan District, Tianjin, 300060 China

**Keywords:** Dexmedetomidine, Epilepsy, Survival, ICU, MIMIC)-IV database

## Abstract

**Background:**

Dexmedetomidine (Dex), midazolam, and propofol are three distinct sedatives characterized by varying pharmacological properties. Previous literature has indicated the positive impact of each of these sedatives on ICU patients. However, there is a scarcity of clinical evidence comparing the efficacy of Dex, midazolam, and propofol in reducing mortality among people with epilepsy (PWE). This study aimed to assess the impact of Dex, midazolam, and propofol on the survival of PWE.

**Methods:**

The data were retrospectively retrieved from the Medical Information Mart for Intensive Care (MIMIC)-IV database (version 2.0). PWE were categorized into Dex, midazolam, and propofol groups based on the intravenously administered sedatives. PWE without standard drug therapy were included in the control group. Comparative analyses were performed on the data among the groups.

**Results:**

The Dex group exhibited a significantly lower proportion of in-hospital deaths and a markedly higher in-hospital survival time compared to the midazolam and propofol groups (*p* < 0.01) after propensity score matching. Kaplan-Meier curves demonstrated a significant improvement in survival rates for the Dex group compared to the control group (*p* = 0.025). Analysis of Variance (ANOVA) revealed no significant differences in survival rates among the Dex, midazolam, and propofol groups (F = 1.949, *p* = 0.143). The nomogram indicated that compared to midazolam and propofol groups, Dex was more effective in improving the survival rate of PWE.

**Conclusion:**

Dex might improve the survival rate of PWE in the ICU compared to no standard drug intervention. However, Dex did not exhibit superiority in improving survival rates compared to midazolam and propofol.

**Supplementary Information:**

The online version contains supplementary material available at 10.1186/s12883-024-03693-1.

## Background

Epilepsy is a severe neurological disorder that affects diverse age groups [1], with approximately 50 million new cases reported annually worldwide, leading to a substantial burden on global healthcare systems [2]. The annual death toll among epilepsy patients reaches 125,000, with 80% occurring in middle- and low-income countries, as outlined in the Global Burden of Epilepsy Report [2, 3]. Cases of epilepsy can become life-threatening, especially in instances of prolonged seizures, such as status epilepticus (SE), which is associated with systemic deterioration and a high mortality rate [2]. To address these challenges, medical associations have proposed some guidelines for terminating epileptic attacks and preventing recurrence, with antiseizure medications representing the primary treatment modality [1, 4–6]. Sedatives and anesthetics are recommended components of antiepileptic drug regimens in these guidelines [4–6].

Midazolam, a prototypical benzodiazepine, is commonly employed during the acute seizure phase. Despite its recommendation for use in patients with convulsive status epilepticus, previous studies have reported considerable variations in its effectiveness, associated morbidity, and mortality [7, 8]. Propofol, another antiepileptic anesthetic widely used in intensive care units (ICUs) to manage SE by modulating cortical epileptic discharges, is chosen to enhance the prognoses of epilepsy treatment owing to its short elimination half-life [9, 10]. Dexmedetomidine (Dex), an alpha2-adrenoreceptor (α2-AR) agonist, demonstrated efficacy in reducing the duration and frequency of epileptic attacks in rats [11]. Dex exerts a protective role against neural excitotoxicity by activating the brain-derived neurotrophic factor signalling pathway [12]. A recent report also highlights the role of Dex in cerebroprotection during epilepsy surgery [13].

Although previous reports have verified the therapeutic efficacy of these sedatives and anesthetics in people with epilepsy (PWE), the evidence regarding their ability to reduce mortality in PWE remains insufficient [14]. The Propofol versus Dexmedetomidine (PRODEX) trial found no significant differences between Dex and Propofol or midazolam in terms of mortality [15]. However, these findings were questioned regarding their applicability to severe trauma patients [16]. Additionally, it is noteworthy that higher doses of Dex have been associated with increased rates of hypotension and bradycardia [17].

Despite this existing research, there is a scarcity of documented comparisons on the survival time and survival rates between Dex, midazolam, and propofol in PWE. Therefore, the primary objective of this study is to elucidate the differential effects of Dex, midazolam, and propofol on the survival rates of PWE in the ICU. The aim is to identify the most promising medication among these sedatives for improving the prognosis of PWE.

## Methods

### Data source

The Medical Information Mart for Intensive Care (MIMIC) is a large public database encompassing medical and health data records of patients in the ICU at Beth Israel Deaconess Medical Center-Women’s Health Care from 2001 to 2019 [18]. The MIMIC database includes comprehensive demographic information such as patient’s sex, height, religion, and race. Since its development, three major versions of the database have been released: MIMIC-II, MIMIC-III, and MIMIC-IV. The data in this study were collected from three sub-databases of the MIMIC-IV database (version 2.0): mimic-core, mimic-hosp, and mimic-icu. The installation of the MIMIC-IV database relies on Navicat Premium 15 and is classified as a PostgreSQL-type database. Acquisition of records was performed by the certified author Xun Li (certificate number: 36,675,346) with data access granted by Beth Israel Deaconess Medical Center-Women’s Health Care. This study was conducted according to the RECORD guidelines [19], and adhered to the principles of the Declaration of Helsinki. The data is in anonymity to protect the confidentiality of the patients. Patient consent was waived by the ethical committee of Tianjin Huanhu Hospital.

### Subjects

The inclusion criteria were as follows: (1) Patients aged 14 to 100 years; (2) Patients diagnosed with any of the following conditions: epilepsy, epileptiform seizure, epilepsy and epileptic seizures, and seizure. The exclusion criteria included: (1) Patients under 14 years or above 100 years of age; (2) Absence of epilepsy-related diagnoses and anesthesia records; (3) Patients who stayed in the ICU for no more than 24 h. After screening, PWE receiving standard drug therapy were categorized into Dex, midazolam, and propofol groups based on intravenous sedation, while those receiving the combination of these intravenous sedative agents were excluded. Additionally, PWE without standard drug therapy were included in the control group.

### Data acquisition

The patients recorded in MIMIC from 2008 to 2019 were included. The independent variables associated with mortality in PWE in the ICU included age, sex, ethnicity, marital status, platelet count, potassium level, sodium level, anion gap, white blood cell (WBC) count, red blood cell (RBC) count, creatinine level, the use or non-use of propofol, midazolam and Dex, heart failure, brain damage, hypoxia, hypotension, and respiratory disease. SQL statements were employed for querying all table data in the database. Data cleaning and pre-processing were performed using Jupyter Notebook, a Python-based editing tool. The relevant variables were extracted from the MIMIC-IV database using codes in Navicat 15 for PostgreSQL and the MIMIC Code Repository (https://github.com/MITLCP/MIMIC-Code). Given the limited studies on epilepsy in the MIMIC database, variable selection was based on existing literature and clinical experiences [20–24]. The survival probabilities of PWE were plotted by Kaplan-Meier curves.

The Match It package in R language employs Propensity Score Matching (PSM). The method parameter is set to “nearest.” The distance parameter defines the distance measure for matching, utilizing the log-probability distance (“logit”). The replace argument determines whether duplicate matches are permitted; we set it to FALSE, indicating that no duplicate matches are allowed. The calliper parameter establishes the maximum matching distance, exclusively applicable when the method is set to “nearest.” It restricts the maximum distance for matching to prevent matches that are too distant. The default value is 0.05, signifying that the matching distance cannot exceed 0.05; this value is also set manually.

The mean in-hospital survival time refers to the mean survival time that PWE had multiple hospital stays and survived since the day of their first hospital stay. The survival rate was the probability of PWE surviving, which was analysed at 1 month, 1 year and 2 year since the first hospital stay.

### Statistical analysis

Quantitative factors with normal distributions were described using means ± standard deviation (SD), while those with non-normal distribution were described using median and interquartile range (Q1, Q3). Qualitative factors were presented as percentages. Data with less than 5% incomplete information were retained, and the rest were multiplicatively imputed, maintaining data precision to three decimal places. Propensity score matching (PSM) was used to ensure equitable group distribution and minimize bias. Demographic scores were closely matched, with the primary difference being the use of medications. The matched function in the R package was used to estimate propensity scores before matching. After adjusting parameters, the propensity score of each intervention item (treatment, i.e., using the current drug to treat patients) was obtained for PWE. All patients were stratified into treatment and control groups, aligning those with similar propensity scores for other variables. An independent-sample *t*-test was used to evaluate the differences between the treatment and control groups. Cox regression analysis was conducted to estimate the impact of multiple physical indicators (e.g., discrete and continuous variables) on the survival time of PWE. Nomograms, widely used in oncology and medical studies for prognosis assessment, visually presented multifactorial regression analysis results (logistic or Cox regression).

Nomograms simplify complex calculations by graphically mapping the values of multiple predictors, which enable rapid estimation of an unknown variable based on known variable values. A typical Nomogram includes axes representing variables, scales for each axis, and lines facilitating variable value estimation. First, the relevant variables were identified for nomogram construction. Next, the variable values were evaluated based on the nomogram, and vertical lines were drawn to determine corresponding points. Subsequently, the data were computed by summing up points for all variables, locating the total on the vertical lines, and associating it with the predictor for the outcome.

R-studio (version 4.2.0) was used for PSM, Cox regression analysis, and nomogram plot. A significance level of *p* < 0.05 (two-tailed) indicated statistical significance. Variance analysis compared continuous data among Dex, midazolam, and propofol groups. The rank sum test was used to evaluate data with skewed distributions. Counting data were analyzed using the chi-square test or Fisher’s exact test. If the theoretical frequency was below five, Fisher’s exact test was employed.

## Results

### Basic information

**Fig. 1:**
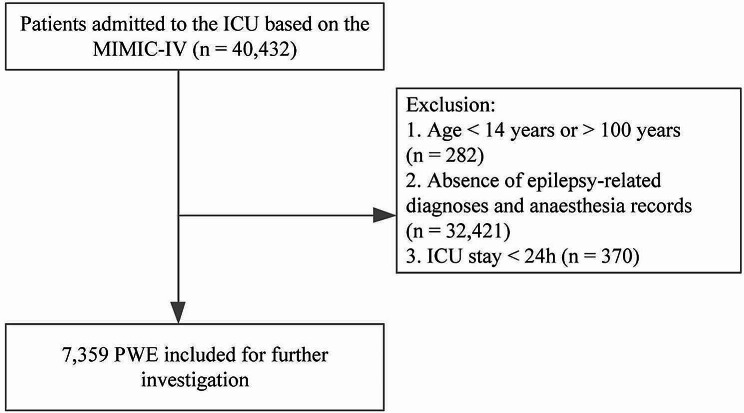
Flow diagram of the present study

The data were obtained from the MIMIC-IV database based on the rigorous inclusion and exclusion criteria (Fig. 1), and a total of 7359 patients were included. These patients were categorized into Dex, midazolam, and propofol groups. Dex, midazolam, and propofol groups had 544 (7.392%), 907 (12.325%), and 1,705 (23.169%) patients, respectively. Among PWE receiving these sedative drugs, there is no statistical differences observed in age, sex, ethnicity, marital status, white blood cells, red blood cells, sodium, creatinine, anion gap, potassium (*p* >0.05, Table 1). Notably, the Dex group exhibited a substantially lower in-hospital death rate (4.1%) compared to both the propofol and midazolam groups (*p* < 0.05, Table 1). Moreover, PWE in the Dex group enjoyed a significantly prolonged mean in-hospital survival time (305 days) in contrast to the propofol (199 days) and midazolam (170 days) groups (*p* < 0.05, Table 1). These compelling outcomes underscore the superior safety profile of Dex for PWE, boasting the highest observed survival rate (95.95%). Propofol, despite being the most frequently employed sedative, demonstrated moderate safety in treating PWE (survival rate: 92.08%), followed closely by midazolam (survival rate: 90.39%).

**Table 1:** Baseline characteristics and survival rate of PWE in the ICU

	Total	Dexmedetomidine	Midazolam	Propofol	*p*-value	
**Age, years**	55.4 ± 18.1	56.0 ± 16.2	57.0 ± 16.2	57.8 ± 16.6	0.0629	
**Sex, n(%)**					0.5477	
Male	3530(47.9)	309(56.8)	489(54.0)	945(55.4)		
Female	3829(52.1)	235(43.2)	418(46.0)	760(44.6)		
**Ethnicity, n(%)**					0.8775	
White	5160(70.2)	325(59.7)	540(59.5)	1030(60.4)		
Black	1033(14.0)	76(13.9)	133(14.6)	255(13.1)		
other	1166(15.8)	143(26.4)	234(25.7)	450(26.3)		
**Marrital_Status, n(%)**					0.9988	
married	2395(32.5)	178(32.7)	306(33.7)	601(35.3)		
other	4964(67.5)	366(67.3)	601(66.3)	1104(64.7)		
**Platelet**	248.3 ± 96.7	224.1 ± 100.3	234.5 ± 105.9	234.7 ± 103.3	0.0078	
**White_Blood_Cells**	8.96 ± 5.78	10.5 ± 8.0	10.6 ± 8.5	10.2 ± 6.8	0.6889	
**Red_Blood_Cell**	4.1 ± 0.7	4.1 ± 0.7	4.1 ± 0.7	4.1 ± 0.7	0.196	
**Sodium**	139.1 ± 4.3	139.1 ± 4.3	139.1 ± 4.4	139.1 ± 4.2	0.9562	
**Creatinine**	1.2 ± 1.3	1.2 ± 1.1	1.3 ± 1.4	1.2 ± 1.4	0.4528	
**Anion_Gap**	14.8 ± 4.4	14.7 ± 4.4	15 ± 4.8	14.6 ± 4.2	0.1947	
**Potassium**	4.1 ± 0.6	4.1 ± 0.6	4.1 ± 0.5	4.1 ± 0.5	0.8435	
**heart_failure, n(%)**					0.0057	
yes	1264(17.1)	178(32.7)	316(34.6)	493(28.9)		
no	6095(82.9)	366(67.3)	591(65.4)	1212(71.1)		
**brian_damage, n(%)**					0.0003	
yes	141(1.9)	21(3.8)	77(8.4)	89(5.2)		
no	7218(98.1)	523(96.2)	830(91.6)	1616(94.8)		
**hypoxia, n(%)**					0.0001	
yes	802(10.8)	268(49.2)	299(32.9)	546(32.1)		
no	6557(89.2)	276(50.8)	608(67.1)	1159(67.9)		
**hypotension, n(%)**					0.0357	
yes	1450(19.8)	210(38.6)	342(37.7)	574(33.6)		
no	5909(80.2)	334(61.4)	565(62.3)	1131(66.4)		
**respiratory, n(%)**					0.0001	
yes	1809(24.5)	385(70.7)	681(75.1)	1046(61.3)		
no	5550(75.4)	159(29.3)	226(24.9)	659(28.7)		
**Time, Median (Q1, Q3)**	157 (4,1296)	383 (24.5,1812.5)	531 (28,1892)	414 (23,1705)	0.0235	
**ratio of in-hospital deaths (%)**	861 (20.5)	22 (4.1)	87 (9.6)	135 (7.9)	0.0021	
**Survival (%)**	3,346 (80)	521 (96)	818 (90)	1,569 (92)	0.0323	
**Average survival time (day)**	/	305	170	199	0.0434	

### Dex improves average in-hospital survival, followed by midazolam and propofol

**Fig. 2:**
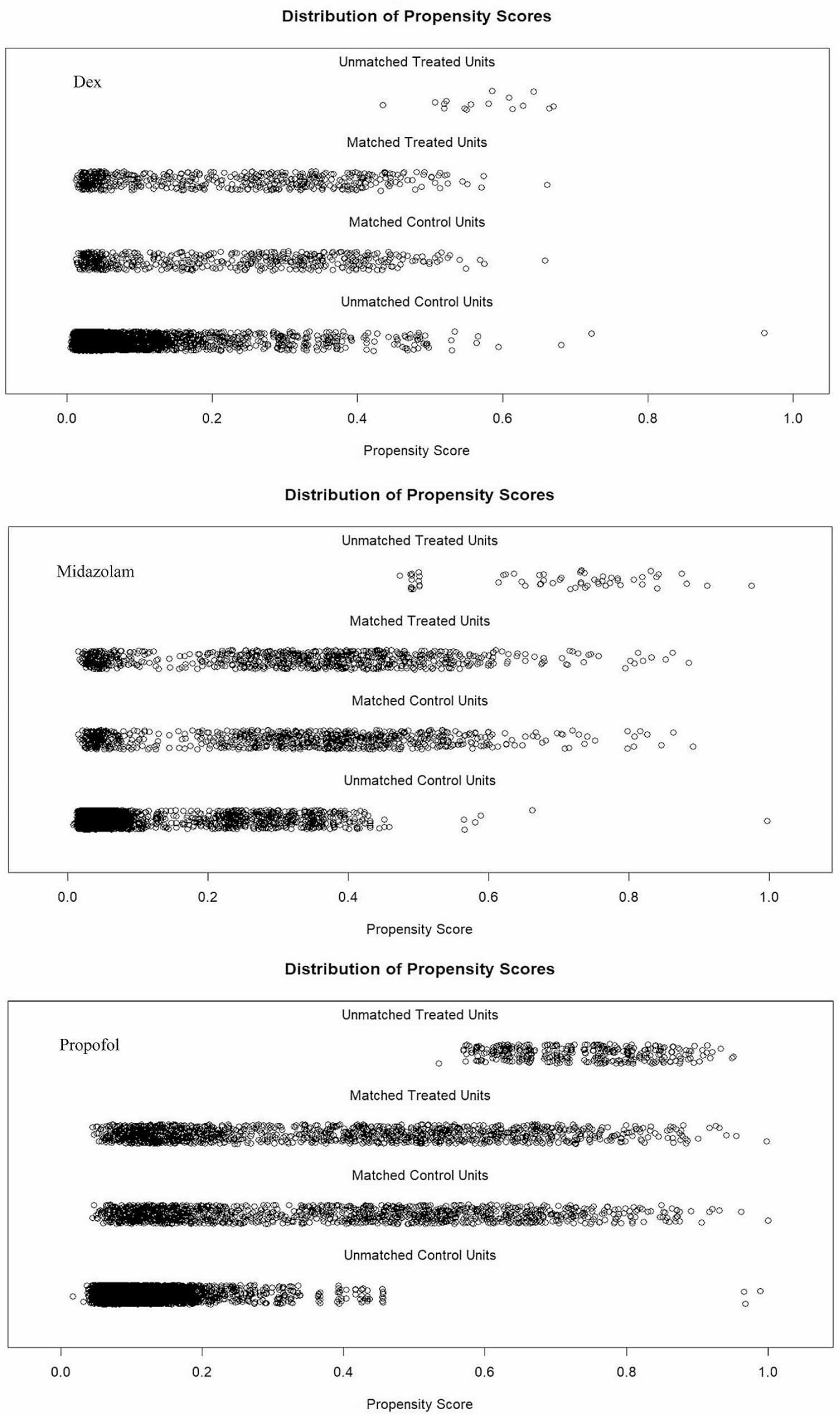
Propensity score matching in the control and treated units with matched and unmatched processes. The matched control and treated units achieved satisfactory compatibility for further analysis

Following PSM, no significant differences were observed in the general clinical characteristics, such as sex, age, blood cell count, and in-hospital death, between the treatment and control groups (*p* > 0.05). The indicators for medication use also showed no variation between the groups. In cases where patients administered no medication with similar parameters were absent, those who received medication were excluded from the analysis. After reviewing the indicators (e.g., age, sex, ethnicity, marital status, platelet count, potassium level, sodium level, anion gap, white blood cell (WBC) count, red blood cell (RBC) count, creatinine level, the use or non-use of propofol, midazolam, and Dex, heart failure, brain damage, hypoxia, hypotension, and respiratory disease) and patient data, both non-homogenous treatment and control groups were established for further analysis (Fig. 2).

The data was processed with reference to the propensity score derived from Raw Treated during the screening of matching results, without reintegrating it. Notably, the propensity-matched dataset exhibited greater relevance and homogeneity compared to its counterpart with Raw Treated propensity score distribution. This pattern is similarly observed for Matched Control and Raw Control. Balance checking was then conducted using Standardized Mean Difference (SMD) measures in the treatment and control groups post-PSM. Generally, a variable is considered to have acceptable trim quality if its SMD does not exceed 0.2. Our results indicated that all SMD values for the examined variables were consistently below 0.2. Detailed findings are provided in the Supplementary file: PSM balance analysis.

The average in-hospital survival days for PWE in the ICU among the Dex, midazolam, and propofol groups were 334, 169, and 225 days, respectively. Conversely, for PWE in the ICU without medication, the average in-hospital survival days in the Dex, midazolam, and propofol groups were 48, 194, and 152 days, respectively. Significant differences in average in-hospital survival were observed between the three treatment and control groups using the independent samples *t*-test (*p* < 0.05). When gender was excluded as an important variable, Dex significantly improved the average survival time of PWE (Dex: 318 days vs. Propofol: 243 days vs. Midazolam: 143 days, *p* < 0.05). Similarly, when age was excluded as an important variable, Dex exhibited a significant improvement in the average survival time of PWE (Dex: 304 days vs. Propofol: 184 days vs. Midazolam: 193 days, *p* < 0.05).

Patients with epilepsy who succumbed in the hospital were screened for medications as an intervention variable, length of survival as an observed variable, and other variables, excluding in-hospital death and medications, as confounding variables. The data from patients exhibiting high homogeneity were matched based on propensity scores.

### Dex, midazolam, and propofol have consistent effects in improving the survival rates of PWE

Cox regression analysis revealed that the platelet count and creatinine level did not exhibit a strong association with the survival time of PWE (*p* > 0.05, Table 2). Although the administration of Dex was not significantly correlated with survival status in PWE based on the univariate regression analysis, the multifactorial analysis revealed a large bias correlation coefficient with the interaction of other indicators, indicating the significant effect of Dex on the survival time of PWE. Similarly, the effects of midazolam and propofol on the survival time of PWE were also significant. Nevertheless, the coefficient for Dex is negative, signifying a decrease in the risk of death with an increase in Dex. Conversely, the coefficients for midazolam and propofol are positive, indicating an increase in the risk of death with higher doses of midazolam and propofol. Considering the absolute values of the coefficients, the hierarchy of the risk of death is as follows: propofol > midazolam > Dex.

**Table 2:** Comparison of characteristics and partial regression coefficient of each factor in PWE

Factors	Coef	Exp (coef)	SE	z	Pr (>|z|)	significance
Age	0.046	1.047	0.005	9.776	< 0.001	***
Sex	0.324	1.383	0.147	2.235	0.025	.
Ethnicity	0.474	1.607	0.080	6.099	< 0.001	***
Marital Status	-0.417	0.659	0.152	-2.752	0.006	*
Dexmedetomidine	-0.804	0.448	0.239	-3.411	< 0.001	**
Midazolam	0.640	1.897	0.167	4.026	< 0.001	*
Propofol	1.041	2.831	0.173	6.385	< 0.001	***
Platelets	<-0.001	0.999	0.000	-1.391	0.164	***
WBCs	0.018	1.018	0.003	5.628	< 0.001	***
RBCs	-0.576	0.562	0.100	-5.930	< 0.001	***
Sodium	0.029	1.029	0.016	1.823	0.068	*
Creatinine	-0.116	0.891	0.061	-2.149	0.032	**
Anion Gap	0.109	1.115	0.015	7.737	< 0.001	***
Potassium	-0.045	0.956	0.125	-0.478	0.632	***
Heart Failure	-0.487	0.614	0.162	-3.010	0.003	**
Brain Damage	1.078	2.939	0.213	5.053	0.000	***
Hypoxia	0.127	1.136	0.172	0.740	0.459	***
Hypotension	-0.688	0.503	0.178	-3.854	0.000	***
Respiratory Disease	0.942	2.564	0.1939	4.856	0.000	***

WBCs: white blood cells; RBCs: red blood cells;

Signif. codes: 0 ‘***’ 0.001 ‘**’ 0.01 ‘*’ 0.05 ‘.’ 0.1 ‘ ’ 1

**Fig. 3:**
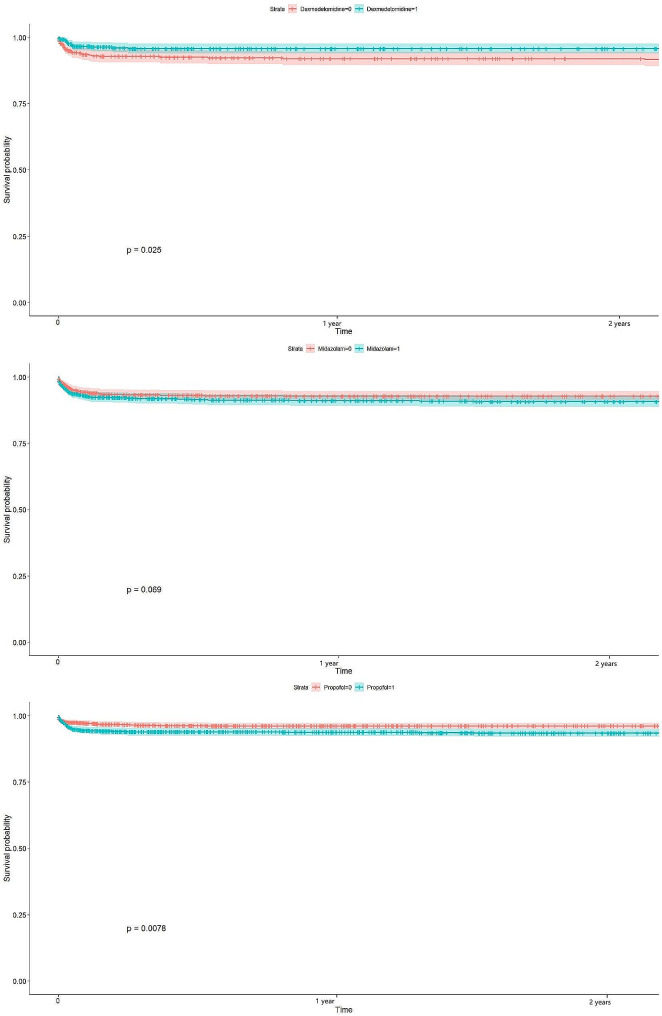
The 2-year survival analysis after propensity score matching. After propensity score matching, Kaplan-Meier curves in the Cox regression analysis revealed a significantly improved survival rate in the Dex group and propofol group compared to the matched control group after a two-year admission

The Kaplan-Meier curves in the Cox regression model of the treatment and control groups are presented in Fig. 3. When compared to the control group, patients in the Dex group exhibited a significant improvement in survival (*p* = 0.025). This trend was not observed in the midazolam in contrast to the control groups (Fig. 3). However, a comparison of the Dex, midazolam, and propofol groups by ANOVA test did not reveal a significant difference (F = 1.949, *p* = 0.143). Thus, compared to midazolam and propofol, Dex was not superior in improving the survival rate of PWE.

### Dex improves the survival rates of PWE, followed by midazolam and propofol

**Fig. 4:**
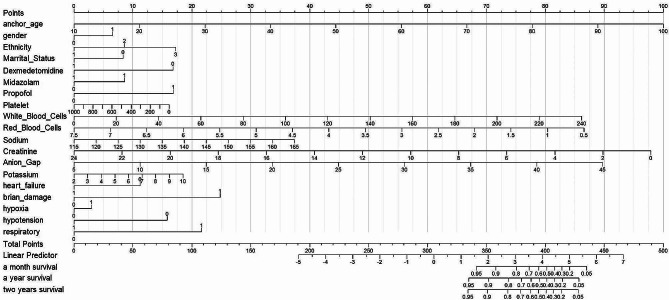
Nomogram of the factors involved in people with epilepsy

In Fig. 4, a nomogram plot depicting the results of the Cox multifactor regression analysis was presented using R-studio for all indicators. Notably, distinct differences in scales for Dex, midazolam, and propofol were evident. The Dex scale at 1 corresponded to 0 points, while scale at 0, it ranged between 16 and 17 points. The midazolam scale equated to 0 points at a scale of 0 and varied between 8 and 9 points at the scale of 1. Similarly, the propofol scale scored 0 points at a scale of 0 and ranged between 16 and 17 points at a scale of 1. The 1-month, 1-year, and 2-year survival rates are presented as total points, with higher points indicating a lower probability of PWE survival. The nomogram indicated that the effectiveness of the three drugs in improving the survival rate of PWE follows the order Dex > midazolam > propofol, which is consistent with the aforementioned results of multifactorial Cox regression analysis.

## Discussion

This study uncovered that Dex significantly enhanced the survival time of PWE compared to the control group. PSM revealed that PWE with similar medication indicators had prolonged survival compared to those without standard drug therapy, with Dex administration leading to the most substantial increase in survival time, followed by propofol and midazolam. Cox multifactor regression analysis, supported by Kaplan-Meier curves, demonstrated that Dex exhibited a significant improvement in survival compared to the control group. However, variance analysis did not reveal any statistical significance among the three treatment groups. The reason for the insignificance of the results may be the uneven distribution of the sample size of the data itself, high data variability, and large variability (variance) among data points in the present clinical database. Nomogram plots indicated that Dex contributed less to the score than propofol and midazolam, with a higher total score representing a lower probability of survival during the assessment period. Therefore, our findings suggest that Dex plays a more significant role in enhancing the survival rate, followed by midazolam and propofol.

Although several studies have assessed the effects of Dex on various diseases, limited evidence exists regarding its impact on the survival of PWE. Cetindag Ciltas et al. [11] reported the anticonvulsant properties of 0.1 mg/kg Dex in a murine epilepsy model, demonstrating its protective effects against pentylenetetrazol-induced seizures. Similarly, another study using kainic acid-induced epilepsy models highlighted the neural protection role of Dex via reducing MAPK phosphorylation and increasing brain-derived neurotrophic factor expression in the hippocampus [12]. Despite these insights into the protective effects of Dex, its potential to attenuate epileptic discharges remains unclear. Dex is preferred by neurosurgeons during epilepsy surgery due to its minimal effects on interictal spike activity and lack of alterations in the electroencephalogra [25–27]. In addition, Dex has no major adverse effects during epilepsy surgery [25–27], which may be due to the specific pharmacologic action of Dex on the subcortical areas of the central nervous system, without interfering with GABA receptors, resulting in sedation resembling natural sleep [28]. The safe use of Dex during epilepsy surgery, with insignificant effects of Dex on hemodynamic indices within an acceptable range, adds to its appeal [28, 29]. Another study reported the safe use of Dex in children with epilepsy, accelerating the sedation rate [30]. Although current studies offer diverse perspectives on the survival-facilitating impacts of Dex in patients with severe brain injuries, more extensive research is needed to evaluate its real-world benefits in PWE.

Limited studies have investigated and compared the prognostic potential of Dex, midazolam, and propofol in PWE in the ICU. A study conducted in India [31] compared seizure control and complications associated with propofol infusion to those associated with midazolam for treating refractory status epilepticus (RSE). This prospective randomized study reported an overall incidence of super RSE at 69.5%, with no significant differences in complication rates between the two groups [31]. Another clinical cohort study involving 386 patients found comparable outcomes between those managed with propofol or midazolam [32]. Therefore, the choice of anesthetic did not significantly affect the overall prognosis of RSE. A systematic review and meta-analysis compared the efficacy of propofol with midazolam in sedated adult ICU patients, evaluating parameters such as ICU stay time, mechanical ventilation time, and extubation time [33]. The results showed that propofol reduced mechanical ventilation time by 4.46 h and extubation time by 7.95 h compared to midazolam [33]. Heybati et al. [34] compared the effects of sedation with Dex and propofol in adult ICU patients on mechanical ventilation, evaluating the major outcomes such as the length of ICU stay, mechanical ventilation duration, ICU delirium, all-cause mortality, and hemodynamic effects. They reported that Dex did not significantly affect ICU stays compared to propofol, but significantly reduced mechanical ventilation duration and delirium risk for heart surgery patients [34]. Another study favored the propofol-dexmedetomidine combination over the propofol-remifentanil combination for conscious sedation in epilepsy surgery due to its fewer side effects [35]. However, there is a notable gap in research comparing the efficacy of Dex and midazolam specifically in PWE. In a systematic review of clinical trials across different populations, Dex exhibited higher operator and patient satisfaction than midazolam, with similar safety profiles [36]. Variable conclusions in different studies may be attributed to selection bias and differences in target populations. Our findings, as indicated by both nomogram and regression analysis, demonstrate that Dex is effective in improving the survival rate. However, the Kaplan-Meier curve did not reveal any discernible differences among the three groups, suggesting that Dex, at least, does not exhibit a significant interaction with the other two groups. It is noteworthy that while Dex, midazolam, and propofol exert varying effects on survival rate, statistical differences may not be evident. It is crucial to interpret this analysis in the broader context, emphasizing that the absence of statistical significance does not diminish the overall value of the observed trends. In results, Dex may not outperform midazolam and propofol in improving the survival rate suggest in terms of the overall efficacy of these sedatives for PWE in the ICU, which shows that patients with severe epilepsy should not be overly concerned about the dangers of sedatives.

While this study sheds light on the potential survival-promoting role of Dex for PWE in the ICU, it has some limitations. Firstly, the absence of long-term follow-up data for survivors from the MIMIC-IV database hinders a comprehensive understanding of their outcomes. Secondly, the limited sample size of patients administered Dex, midazolam, and propofol with a diagnosis of epilepsy complicates subgroup analysis, making it challenging to assess the impact of different seizure types or epilepsy syndromes with diverse etiologies on PWE survival in the ICU. Thirdly, the present study did not assess the effects of different combinations of sedative agents on PWE outcomes, as the small sample size restricts detailed analysis. Finally, the presence of unobserved variables may influence the validity of our results, and potential sources of bias, confounding factors, or other constraints that might impact the generalizability of the results. Further large-scale clinical trials on PWE in the ICU should be encouraged to elucidate the roles of Dex, midazolam, and propofol by incorporating more detailed confounders. Additionally, further analysis is needed to explore whether major deaths in ICU patients are significantly associated with drugs.

## Conclusion

In conclusion, our study suggests that Dex may enhance the survival rate of PWE in the ICU compared to no medical intervention. However, Dex does not appear to outperform midazolam and propofol in improving the survival rate.

### Electronic supplementary material

Below is the link to the electronic supplementary material.

Supplementary Material 1

## Data Availability

No datasets were generated or analysed during the current study.
