# Supplementary material for: Comparative analysis of dexmedetomidine, midazolam, and propofol impact on epilepsy-related mortality in the ICU: insights from the MIMIC-IV database
Source: BMC Neurol. 2024 Jun 7;24:193. doi: 10.1186/s12883-024-03693-1 (PMC11157909; doi:10.1186/s12883-024-03693-1)
Supplement: Supplementary file 1 — Supplementary Material 1 [file 12883_2024_3693_MOESM1_ESM.docx]

### Propensity Score Matching Balance analysis：

The comparison of balance can be measured by the SMD values of the treatment and control group indicators after PSM. Standardized Mean Difference (SMD), also known as Cohen's d, is a commonly used effect size measure to compare differences in the mean between two groups or conditions. SMD can reflect the size of the difference between the means of two sets of data relative to their standard deviations. The greater the absolute value of SMD, the greater the difference between the mean values of the two groups of data. In general, a common criterion for judging is: SMD less than 0.2 indicates a small effect, 0.2 to 0.5 indicates a moderate effect, and greater than 0.5 indicates a large effect.

After PSM analysis, the SMD of Dex、Mid、Pro are shown as below:

| Indicators | Dex_SMD | Mid_SMD | Pro_SMD |
| --- | --- | --- | --- |
| anchor_age | -0.024555618 | -0.035707083 | -0.047661425 |
| gender | -0.038199916 | 0.002364009 | -0.028362258 |
| Ethnicity | 0.051271577 | 0 | -0.028050867 |
| Marrital_Status | 0.073849987 | -0.054748289 | -0.032630465 |
| Platelet | 0.001476563 | -0.033395032 | 0.01923534 |
| White_Blood_Cells | 0.047482362 | 0.040451164 | 0.015487821 |
| Red_Blood_Cells | -0.01712817 | 0.002608821 | 0.018788367 |
| Sodium | 0.066173668 | -0.038725208 | 0.032904786 |
| Creatinine | 0.042123697 | 0.04442869 | -0.000355843 |
| Anion_Gap | 0.00092462 | 0.031829696 | 0.01372674 |
| Potassium | 0.032039611 | 0.002834261 | 0.016779322 |
| heart_failure | -0.07569281 | -0.034746149 | -0.020351507 |
| brian_damage | -0.028086104 | 0.022241856 | 0.056565451 |
| hypoxia | 0.007571073 | -0.057508902 | 0.050657339 |
| hypotension | 0.003911622 | -0.060976644 | -0.026079772 |
| respiratory | -0.008258163 | 0 | 0.020822371 |
| distance | 0.001376879 | 0.004107112 | 0.014023137 |

In the results shown in the table above, the absolute value of SMD for all indicators is less than 0.2, so the data are balanced between the three-drug treatment group and the control group after matching by PSM propensity score.
